# The amount of activating EGFR mutations in circulating cell-free DNA is a marker to monitor osimertinib response

**DOI:** 10.1038/s41416-018-0238-z

**Published:** 2018-11-06

**Authors:** Marzia Del Re, Paola Bordi, Eleonora Rofi, Giuliana Restante, Simona Valleggi, Roberta Minari, Stefania Crucitta, Elena Arrigoni, Antonio Chella, Riccardo Morganti, Marcello Tiseo, Iacopo Petrini, Romano Danesi

**Affiliations:** 1 Clinical Pharmacology and Pharmacogenetics Unit, Department of Clinical and Experimental Medicine, University of Pisa, Pisa, Italy; 2 Medical Oncology Unit, University Hospital of Parma, Parma, Italy; 3 Pneumology Unit, Department of Translational Research and New Technologies in Medicine, University of Pisa, Pisa, Italy; 4 Section of Statistics, Department of Clinical and Experimental Medicine, University of Pisa, Pisa, Italy

**Keywords:** Non-small-cell lung cancer, Predictive markers

## Abstract

**Background:**

Circulating cell-free DNA (cfDNA) may help understand the molecular response to pharmacologic treatment and provide information on dynamics of clonal heterogeneity. Therefore, this study evaluated the correlation between treatment outcome and activating EGFR mutations (act-EGFR) and T790M in cfDNA in patients with advanced NSCLC given osimertinib.

**Methods:**

Thirty-four NSCLC patients resistant to first/second-generation EGFR-TKIs, positive for both act-EGFR and T790M in cfDNA at the time of progression were enrolled in this study. Plasma samples were obtained at osimertinib baseline and after 3 months of therapy; cfDNA was analyzed by droplet digital PCR and results were expressed as mutant allele frequency (MAF).

**Results:**

At baseline, act-EGFR MAF was significantly higher than T790M (*p* < 0.0001). act-EGFR MAF and T790M/act-EGFR MAF ratio were significantly correlated with disease response (*p* = 0.02). Cut-off values of act-EGFR MAF and T790M/act-EGFR ratio of 2.6% and 0.22 were found, respectively. The PFS of patients with act-EGFR MAF of > 2.6% and < 2.6%, were 10 months vs. not reached, respectively (*p* = 0.03), whereas patients with T790M/act-EGFR ≤ 0.22 had poorer PFS than patients with a value of > 0.22 (6 months vs. not reached, respectively, *p* = 0.01).

**Conclusion:**

act-EGFR MAF and T790M/act-EGFR MAF ratio are potential markers of outcome in patients treated with osimertinib.

## Background

Approximately 50% of non-small cell lung cancer (NSCLC) patients with activating mutations of epidermal growth factor receptor (act-EGFR), who progress to first- (gefitinib, erlotinib) or second-generation (afatinib) EGFR tyrosine kinase inhibitors (EGFR-TKIs), acquire drug resistance due to the T790M mutation^1^, which changes a conserved threonine to methionine in EGFR.^2^ Indeed, T790M is located within the gatekeeper region and, as methionine is larger than threonine, it prevents the interaction of early-generation EGFR-TKIs with the ATP-binding site^3^, whereas the affinity to ATP is conserved or increased.^4^ Osimertinib is a third-generation EGFR-TKI strongly active against T790M-positive NSCLC.^5^ Its use is allowed in T790M-positive NSCLCs tested in tissue biopsy or in plasma.^6,7^ Although T790M is rarely detected at diagnosis,^8,9^ its assessment is mandatory at disease progression (PD) after treatment with first-line EGFR-TKIs. Therefore, to avoid repeated, invasive, and sometimes technically difficult tissue biopsies, the analysis of circulating cell-free DNA (cfDNA) is accepted as an alternative approach for molecular analysis where tumour biopsy is not feasible.^10^ cfDNA analysis also allows real-time monitoring of the clonal evolution, which involves primary tumours and metastatic sites as well.^11^ Indeed, studies assessing the efficacy of osimertinib in T790M-positive patients showed that cfDNA can be used as a surrogate marker for T790M in tumour tissue.^7^ The knowledge regarding the kinetics of act-EGFR during osimertinib treatment is still based on preliminary findings^12^ and cfDNA could be a valuable tool to better understand tumour evolution, helping us identify predictive biomarkers of response. In the present study, we describe the changes of act-EGFR and T790M in cfDNA in relation to clinical outcome of patients treated with osimertinib and found that the amount of act-EGFR, but not of T790M, at baseline and during the treatment is a potential biomarker of response of patients treated with osimertinib.

## Patients and methods

A total of 34 NSCLC patients, taking part to the ASTRIS trial (NCT02474355), were enrolled in this study. Subjects must have (1) primary tumours positive for act-EGFR (exon 19 deletion [ex19del], exon 21 L858R, or other mutations [i.e., L861Q]), (2) PD after first- or second-generation EGFR-TKIs (gefitinib, erlotinib, or afatinib) associated with detectable T790M at liquid and/or tissue re-biopsy. Tissue analysis for act-EGFR (at diagnosis and, if available, at progression) and T790M (at progression, if available) was done by standard diagnostic procedures in use in each centre (i.e., EGFR TKI response®, Diatech, Jesi, Italy; Therascreen®, Qiagen, Valencia, CA). An additional requirement to be enrolled in this study was the presence of act-EGFR and T790M in cfDNA at baseline, as defined below.

Progression-free survival (PFS) is the time from assignment to treatment to PD or death from any cause. Complete (CR) and partial responses (PR), stable disease (SD), and PD are defined as per RECIST criteria v. 1.1 and were assessed at 3 months of treatment. Disease control rate (DCR) is defined as the percentage of patients who achieved CR, PR, and SD, while overall response rate (ORR) is the cumulative proportion of patients who have CR or PR.

### Plasma sampling, cfDNA extraction, and analysis

Plasma samples for the analysis of cfDNA were taken before the first dose of osimertinib (baseline) and at the first clinical evaluation (after 3 months). Six milliliter of blood were collected in EDTA tubes and centrifuged for 10 min at 3000 r.p.m. at room temperature within 2 h after blood drawing. Full details of the method have been previously published.^13^ Briefly, cfDNA was extracted using a QIAmp Circulating Nucleic Acid kit (Qiagen®) from 3 ml of plasma and the DNA was eluted in 100 μl of buffer. The analysis of cfDNA was performed by digital droplet PCR (ddPCR) using the ddPCR Mutation Assay (BioRad®, Hercules, CA). A fluorescence intensity threshold of 3000 was set as a cut-off point; the sample was considered as act-EGFR and T790M positive when at least one droplet was above the threshold level. act-EGFR and T790M values were reported as mutant allele frequency (MAF), defined as the proportion of mutant to wild-type PCR products in the ddPCR readout; T790M/act-EGFR MAF ratio was also calculated. Patients with PD at first assessment (3 months) underwent tissue biopsy, if feasible, and cfDNA C797S analysis, in addition to act-EGFR and T790M, to investigate the reason of resistance to osimertinib.

### Statistical analysis

Before performing inferential analysis, an exploratory phase was carried out. To evaluate the normality of the quantitative data distributions, the Kolmogorov–Smirnov test was performed. The assessment of the paired data (matched and repeated) was performed by Wilcoxon’s test (two-tailed), whereas the evaluation of independent samples was performed with Kruskal–Wallis and Mann–Whitney (two-tailed) tests. Predictive value of T790M/act-EGFR MAF ratio at baseline was determined by receiver-operating curve (ROC) analysis and area under curve was assessed by a non-parametric test; the best cut-off was also calculated applying the Youden index. The dichotomous ratio calculated after ROC analysis was impacted with the PFS by Cox regression and the results were expressed by hazard ratios with confidence interval 95% and related *p*-value. Differences were considered significant at *p* < 0.05. All analyses, descriptive ad inferential, were carried out using the SPSS v.24 technology.

## Results

Table 1 summarises the demographic characteristics of patients. The act-EGFRs in tissues at diagnosis were ex19del in 25 subjects (73.5%), L858R in 8 patients (23.5%), and L861Q (3%) in 1 patient; interestingly, in this subject, ex19del was also found only in cfDNA at baseline and during the follow-up.

**Table 1:** Characteristics of patients

**Number of patients**	34
**Age (years, range)**	63 (42–81)
**Sex**	
Male	10 (28.6%)
Female	24 (71.4%)
**Smoking history**	
Former	11 (32.3%)
Never	23 (67.7%)
**Stage**	
Stage IV	32 (94.2%)
Stage IIIb	2 (5.8%)
**Prior EGFR-TKIs**	
Gefitinib	21 (61.8%)
Erlotinib	8 (23.5%)
Afatinib	5 (14.7%)
**Lines of treatment before osimertinib**	
First-line	13 (38.2%)
>1 line	21 (61.8%)

All patients received a prior EGFR-TKI and 21 patients received > 1 line of therapy. The response rate to osimertinib in this population was 50%, with a DCR of 76.5% and a median PFS of 9.9 months; 8 patients showed PD at first evaluation.

**Fig. 1:**
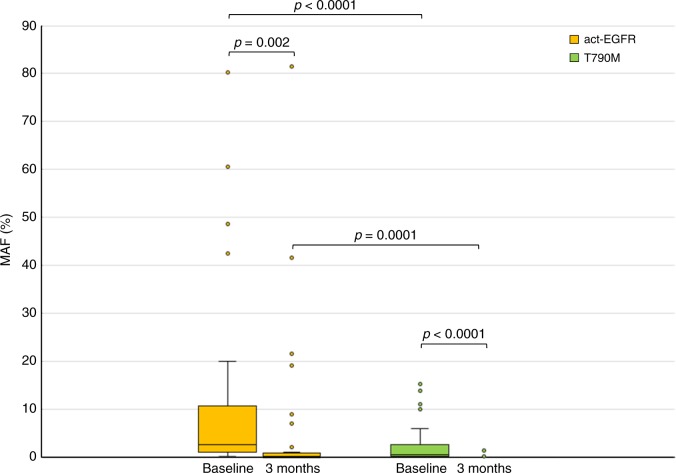
Act-EGFR and T790M MAF at baseline vs. 3 months. Data are expressed as MAF (%) (outliers excluded)

**Fig. 2:**
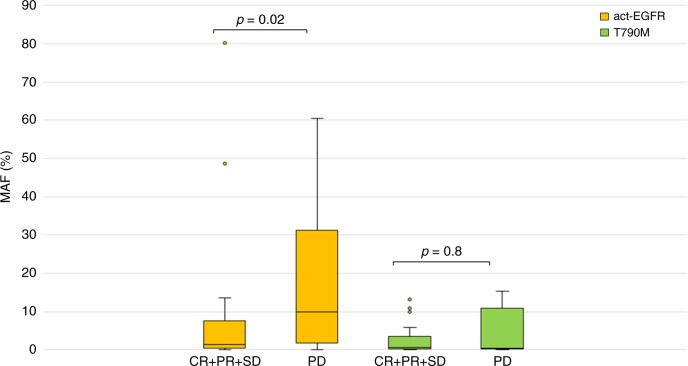
Act-EGFR MAF in patients achieving CR/PR/SD vs. PD. Data are expressed as MAF (%) and range

At baseline, the median act-EGFR MAF (2.6%) was significantly higher than T790M (0.575%, *p* < 0.0001; Fig. 1). Act-EGFR MAF was related to disease control (*p* = 0.02; Fig. 2), whereas T790M MAF was not (*p* = 0.8; Fig. 2). Act-EGFR and T790M MAFs were related to previous lines of treatment; in particular, patients who received >  1 line of therapy had higher act-EGFR and T790M MAFs compared with patients who received one line of therapy (act-EGFR 6.2% vs. 1%, *p* = 0.01; T790M 0.6% vs. 0.2%, *p* = 0.05). No correlations were found between act-EGFR and T790M MAFs, and the number of tumour sites.

**Fig. 3:**
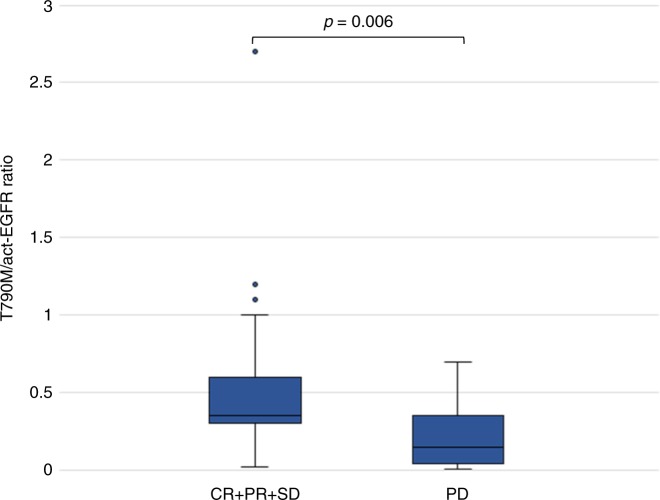
T790M/act-EGFR ratio in patients achieving CR/PR/SD vs PD. Data are expressed as T790M/act-EGFR ratio

The median T790M/act-EGFR MAF ratio at baseline was 0.28 in the overall population and 0.35 vs. 0.18 in patients who achieved a disease control vs. PD, respectively (*p* = 0.006) (Fig. 3).

In the overall population, act-EGFR (2.6% to 0.2%, *p* = 0.002) and T790M (0.575% to 0%, *p* < 0.0001) were significantly decreased from baseline to 3 months (Fig. 1). A disappearance or strong reduction of both act-EGFR and T790M MAFs were observed after 3 months in responding patients (CR + RP) and also in patients with SD, although the decline of act-EGFR was less pronounced compared with T790M (*p* = 0.002 and *p* < 0.0001, respectively) (Table 2).

**Table 2:** Median (X) and range of act-EGFR and T790M MAFs at baseline and at first tumour assessment (3 months), ratio of T790M/act-EGFR at baseline and tumour response in patients treated with osimertinib

Patient	act-EGFR		T790M		Ratio	Response
	Baseline	3 Months	Baseline	3 Months		
1	1.5	0.28	0.9	0	0.6	CR
2	2.1	0	0.6	0	0.3	CR
3	8.2	0	0.17	0	0.02	CR
4	12.7	0	3.5	0	0.3	CR
***X*** **(range)**	**5.2 (1.5–12.7)**	**0 (0–0.28)**	**0.8 (0.2–3.5)**	**0 (0–0)**	**0.3 (0.02–0.6)**	
5	48.6	0	11	0	0.2	PR
6	1.3	0.5	0.4	0	0.3	PR
7	9.5	0.41	10	0.2	1.1	PR
8	80.2	0	13.2	0	0.2	PR
9	1	0.22	0.18	0	0.2	PR
10	0.9	0	0.55	0	0.6	PR
11	5.9	0	1.6	0	0.3	PR
12	0.52	1	0.14	0	0.3	PR
13	0.44	0	0.36	0	0.8	PR
14	0.11	0	0.07	0	0.6	PR
15	0.1	0.4	0.12	0	1.2	PR
16	0.6	0	0.2	0	0.3	PR
17	3.4	0	0.15	0	0.04	PR
***X*** **(range)**	**1 (0.1–80.2)**	**0 (0–1)**	**0.4 (0.1 – 13.2)**	**0 (0–0.2)**	**0.3 (0.04–1.2)**	
18	7.7	0.75	0.8	0.5	0.1	SD
19	1.4	0.2	0.6	0	0.4	SD
20	6.2	0.29	5	0	0.8	SD
21	13.7	0	3.6	0	0.3	SD
22	1	0.2	0.6	0	0.6	SD
23	2.2	2.1	5.9	0.8	2.7	SD
24	2.5	0.24	0.94	0	0.4	SD
25	0.5	0.2	0.2	0.1	0.4	SD
26	0.4	0	0.4	0	1	SD
***X*** **(range)**	**2.2 (0.4–13.7)**	**0.2 (0–2.1)**	**0.8 (0.2–5.9)**	**0 (0–0.8)**	**0.4 (0.1–2.7)**	
27	10	21.6	0.3	0	0.03	PD
28	20	81.4	0.16	0	0.008	PD
29	2.9	41.6	0.5	0	0.1	PD
30	60.5	0	13.8	0	0.2	PD
31	42.4	19.1	15.3	0	0.4	PD
32	1	0	0.2	0	0.2	PD
33	2.7	7	0.2	1.3	0.07	PD
34	12.7	9	2.4	0	0.7	PD
***X*** **(range)**	**11.4 (1–60.5)**	**14.1 (0–81.4)**	**0.4 (0.2–15.3)**	**0 (0–1.3)**	**0.2 (0.008–0.7)**	

Eight patients had early PD during osimertinib treatment; in four of them there was a strong increase in act-EGFR MAF (patients 27, 28, 29 and 33), whereas in the other four it was decreased or undetectable (Table 2). T790M disappeared from plasma in all patients but one (patient 33) despite evidence of PD (Table 2). The reason of early resistance was demonstrated on re-biopsy (SCLC transformation in two patients, c-MET amplification one patient, C797S in another patient; Table 3). In the remaining four subjects, tumour biopsy in one and cfDNA in three did not provide evidence of specific mechanism of resistance (Table 3).

**Table 3:** Clinical characteristics of patients progressed to osimertinib

Pt #	Gender	Age	1-line TKI	1-line PFS (mo)	Site of PD during osimertinib	Site of tissutal re-biopsy at PD (if available)	Tissutal re-biopsy result
27	M	56	Gefitinib	25.9	Lung, liver, lymph nodes	Liver	SCLC histology, ex19del-, T790M-
28	M	68	Afatinib	18.5	Adrenal gland	Adrenal gland	SCLC histology, L858R + , T790M-
29	M	52	Erlotinib	12	Liver	Liver	ex19del + , T790M-, MET amplification
30	M	50	Gefitinib	7.8	Liver, soft tissues, lymph nodes	–	–
31	M	71	Afatinib	13.1	Lung, soft tissues, lymph nodes, bones	–	–
32	F	86	Gefitinib	7.1	Lung, pleural effusion	–	–
33	F	42	Afatinib	9.9	Lung, liver, mesenteric, bone, brain	Lung	ex19del + , T790M-, C797S +
34	F	74	Gefitinib	11.2	Lung	Lung	ex19del+, T790M-

**Fig. 4:**
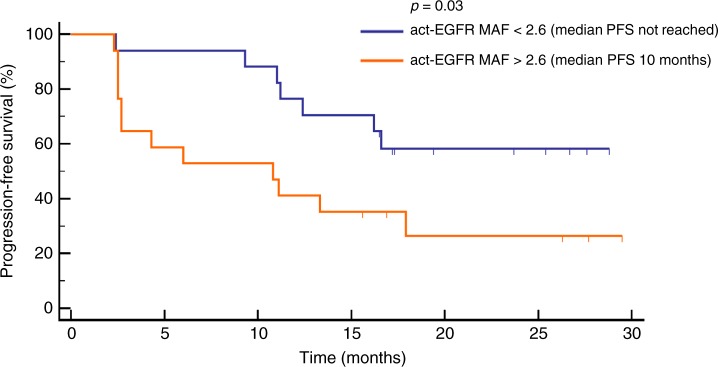
PFS of patients stratified on the basis of cut-off value (2.6%) of act-EGFR MAF calculated by ROC analysis

**Fig. 5:**
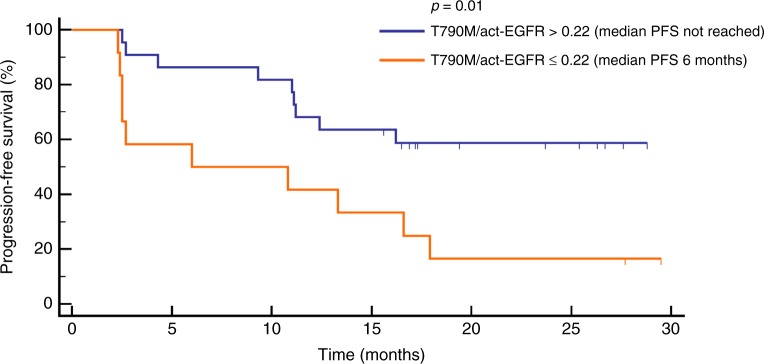
PFS of patients stratified on the basis of cut-off value (0.22) of T790M/act-EGFR ratio calculated after ROC analysis

Finally, a ROC curve analysis was performed to identify the best cut-off values of act-EGFR MAF and T790M/act-EGFR ratio and found, respectively, 2.6% and 0.22. The PFS of patients with act-EGFR MAF of > 2.6% and < 2.6%, were 10 months vs. not reached, respectively (*p* = 0.03; Fig. 4), whereas patients with T790M/act-EGFR ≤ 0.22 had poorer PFS than patients with a value of > 0.22 (6 months vs. not reached, respectively, *p* = 0.01; Fig. 5).

## Discussion

Non-invasive monitoring of tumours provides important information for personalised treatment of NSCLC. As cells harboring the T790M mutation are resistant to first- and second-generation EGFR-TKIs, its detection in tissue or plasma influences the choice of whether to use or not a third-generation EGFR-TKI, such as osimertinib.

ddPCR-based analysis of cfDNA is a promising method and it is acceptable to identify T790M-positive tumours, although patients with T790M-negative cfDNA samples should be tested on tumour tissue obtained by biopsy.^7,14–16^ Osimertinib is designed to target T790M and act-EGFR more selectively than wild type EGFR.^16^ As previously reported by our group^12^ and confirmed in this work, at osimertinib baseline, the amount of the act-EGFR was significantly higher than T790M, indicating that cell clones resistant to first-/second-generation EGFR-TKIs represent a minority among cells bearing act-EGFR, and that they arise from the selective pressure of therapy. The lack of a significant correlation between baseline T790M MAF and disease response is not surprising, as demonstrated by the FLAURA trial (NCT02296125), showing a consistent benefit in terms of PFS of osimertinib vs. standard treatments (gefitinib/erlotinib) in EGFR-TKI-naïve patients not selected for T790M positivity.^17^ These data suggest that osimertinib efficacy is not simply predicted by the presence of T790M. Moreover, other studies did not show a correlation between T790M level and response to osimertinib.^18^ On the contrary, the act-EGFR MAF and the ratio of T790M/act-EGFR at baseline seemed to be reliable markers to predict the benefit of treatment; indeed, the risk of progression to osimertinib is higher in patients with elevated act-EGFR MAF and, therefore, with lower T790M/act-EGFR ratio. Interestingly, the cut-off of 0.22 of the T790M/act-EGFR ratio is able to discriminate patients with longer PFS, with a sensitivity of 81%. The potential predictive role of T790M/EGFR activating ratio was also observed in other studies.^7,19,20^

In general, patients studied in our cohort presented a significant decrease in both act-EGFR and T790M at first evaluation, as compared with baseline. Although in a previous study^21^ a clearance of plasma EGFR mutations at 6 weeks was associated with longer median PFS and better ORR, we did not find the same association in our population, as the majority of our patients had a complete clearance of T790M at 3 months, including all but one of those with PD. On the contrary, act-EGFR decreased in patients responding to treatment, whereas it was increased in four out of eight subjects with PD. Thus, our data suggest that cfDNA analysis of T790M is not useful to monitor response to osimertinib, whereas act-EGFR assessment is significantly associated with disease outcome and thus more informative. These data are in agreement with another publication indicating that T790M may disappear also in patients progressing to osimertinib,^22^ confirming that T790M is not a good biomarker to monitor response and tumour relapse during treatment. A previous case report documented the amplification of EGFR in cfDNA as a mechanism of resistance to osimertinib in a patient with increasing amount of mut-EGFR in cfDNA.^23^

Several mechanisms of resistance to osimertinib have been described, including the EGFR C797S that abolishes the binding of osimertinib to EGFR, as well as G796S/R in addition to a hinge pocket L792F/H mutation.^24^ Furthermore, c-MET amplification,^19,25^ ERBB2, wild-type EGFR somatic copy-number alterations, L798I,^19^ and SCLC transformation^26^ have been documented in patients resistant to third-generation EGFR-TKIs.

The present study has two relevant limitations to consider: the retrospective nature of the analysis and the small sample size. For these reasons, future prospective studies with adequately sample size will be necessary to strengthen the results of the present work.

In conclusion, this proof-of-concept study provides further evidence of the importance of T790M detection in plasma of patients resistant to first/second-generation EGFR-TKIs, in order to switch to osimertinib. However, act-EGFR proved to be a more reliable marker of response/resistance to treatment; thus, monitoring its increase in cfDNA in serial plasma samples strongly suggests the development of resistance in osimertinib-treated patients.
